# Observing life from the sideline – a qualitative study on experiences of living with pregnancy-related pelvic girdle pain

**DOI:** 10.1186/s12884-026-08724-y

**Published:** 2026-02-04

**Authors:** Evelyn Kleppe-Danby, Anne Marie Gausel, Eva Christina Risa Furskog, Cecilia Bergström

**Affiliations:** 1 Department of Obstetrics and Gynaecology, Stavanger University Hospital, Gerd-Ragna Bloch Thorsens gate 4, Stavanger, 4011 Norway; 2 Department for Caring and Ethics, Faculty of Health Sciences, University of Stavanger, Stavanger, Norway; 3 Et Liv I Bevegelse (ELiB), The Norwegian Chiropractic Research Foundation, Oslo, Norway; 4 Department of Clinical Sciences, Obstetrics and Gynaecology, Umeå University, Umeå, Sweden

**Keywords:** Pelvic girdle pain, Lumbopelvic pain, Pregnancy-related pelvic girdle pain, Pregnancy, Qualitative content analysis, Women’s health

## Abstract

**Background:**

Pregnancy-related pelvic girdle pain (PPGP) is a common musculoskeletal condition that affects physical functioning, emotional well-being, and everyday life for many women. While the physical symptoms of PPGP are increasingly recognised, there remains a lack of insight into the lived experiences of those affected, particularly in relation to identity, coping, and family life.

**Aim:**

This study aimed to explore pregnant Norwegian women’s experiences of PPGP, with a particular focus on symptom burden, coping strategies, and the condition’s impact on quality of life and family dynamics.

**Methods:**

Semi-structured in-depth individual interviews (*n* = 18) were conducted with pregnant women experiencing PPGP. Data were analysed using thematic analysis.

**Results:**

The overarching theme, *This*,* too*,* shall pass*, emerged from participants’ narratives, capturing a shared perception of PPGP as a temporary, though challenging, aspect of the perinatal experience. While some participants remained hopeful for immediate postpartum relief, others anticipated a longer recovery. The participants described a constant need to adapt, striving to balance daily responsibilities and self-preservation despite persistent discomfort. PPGP significantly disrupted physical and emotional functioning, disrupted sleep, and hindering routine activities, including work participation. Partner support and social validation emerged as vital coping resources.

**Interpretation:**

Our findings show that PPGP profoundly affects women’s physical, emotional well-being, occupational roles and sense of identity during pregnancy. These insights highlight the need for a holistic, person-centred approach to PPGP in clinical care. Improving professional awareness and the development of patient-informed interventions are essential to support coping strategies, safeguard maternal identity, and reduce the daily burden of living with PPGP.

**Supplementary Information:**

The online version contains supplementary material available at 10.1186/s12884-026-08724-y.

## Introduction

Pregnancy-related pelvic girdle pain (PPGP) is a prevalent and sometimes debilitating condition affecting a substantial proportion of pregnant women. Estimates suggest that 20% to 65% of women experience PPGP at some stage during pregnancy [1–3], with pooled prevalence rates of 63% [4]. While PPGP is a self-limiting condition for most, lasting during pregnancy or shortly postpartum, it can cause debilitating symptoms for others, resulting in persistent pain, significant discomfort, and functional limitations [5–7].

Women affected by PPGP can experience a significant reduction in their ability to perform everyday tasks, such as standing, walking, and turning over in bed [8]. They can also face challenges in fulfilling their roles as mothers, managing household chores, and meeting work-related responsibilities [9, 10]. PPGP not only presents physical challenges but also significantly affects women’s emotional and psychological well-being [11, 12]. Women with PPGP commonly report feelings of frustration and helplessness, which can persist after delivery, particularly when combined by difficulties in performing routine childcare tasks [10, 13]. PPGP is also associated with an increased risk of mood disorders such as anxiety and depression, sleep disturbances, and a diminished quality of life [1, 14–16]. Additionally, PPGP can influence women’s perceptions of future pregnancies, as they may worry about managing the condition while caring for a young child [17].

PPGP has a significant impact on work participation, with many women requiring sick leave and experiencing a sense of loss from being away from the stimulation of work [9, 18, 19]. Studies show that sick leave is higher among women with PPGP than those without PPGP, with over 60% on sick leave by the third trimester [20] thus the economic impact of PPGP in Norway is significant. The estimated loss of person-years of labor for women experiencing temporary or permanent disability due to PPGP amounts to USD 190 millions [21]. Indirect costs, such as productivity losses, work impairment, and absenteeism, account for nearly 90% of the total costs [21].

Despite its prevalence and significant health burden, research on women’s lived experiences with PPGP remains limited. We therefore aimed to address this gap by using qualitative inductive in-depth individual interviews to explore Norwegian women’s experiences of PPGP, focusing on their symptoms, coping strategies, and the condition’s impact on their quality of life and family dynamics.

## Materials and methods

This study is part of the Pelvic Girdle Pain in a NORwegian cohort (PGP-NOR) project, a cohort study investigating PPGP in Norwegian women. The main objective of the PGP-NOR project is to gain a deeper understanding of PGP (pelvic girdle pain) in pregnant women from approximately gestational age (GA) 12 weeks, with an ongoing follow-up until 24-months postpartum. This project is a two-phase study based in western Norway, combining both qualitative and quantitative components. This paper focuses on the qualitative aspects of the study.

### Design

We employed an inductive explorative qualitative interview approach, targeting a diverse group of pregnant Norwegian women experiencing varying degrees of PPGP. This method was chosen for its capacity to uncover unanticipated insights by capturing a broad range of perspectives. By avoiding the imposition of predefined theoretical frameworks, this approach allowed for a comprehensive exploration of overt and nuanced patterns of differences and similarities within the data.

Written information about the study, consent forms, questionnaires, and interview recordings were collected using Nettskjema. Nettskjema, a web-based tool developed and managed by the University Centre for Information Technology at the University of Oslo, was utilised for all aspects of data collection, including consent forms, questionnaires, and interview recordings. This platform enables responses to be submitted from any browser and ensures compliance with Norwegian privacy regulation (GDPR), making it a secure and user-friendly option for online surveys [22].

### The Norwegian setting

Norway, located in Northern Europe, is the 10th wealthiest country in the world [23], with approximately 5.6 million in the last quarter of 2024 [24]. In 2023, there were 52,000 live births [25]. All pregnant women in Norway are offered nine antenatal care (ANC) visits throughout pregnancy, covering blood tests, consultations, glucose testing, and two routine obstetric ultrasounds: one at GA 11–14 and the second in GA 18 [26]. Women can consult a midwife, their general practitioner (GP), or a combination of both at their discretion. Only 0.1% of women giving birth in Norway in 2000, had not received antenatal care during their pregnancy [27]. The Norwegian healthcare system is tax-based, and maternity care is provided free of charge [28].

### Recruitment of participants

Pregnant women attending ANC at five local health centres and those receiving rehabilitation for PPGP at two multidisciplinary clinics in western Norway were invited to participate in the study. All participants had been diagnosed with PPGP by either a midwife or a musculoskeletal specialist clinician. However, there was no requirement for a clinical examination to diagnose PPGP. Women were eligible for inclusion if they met the following criteria: aged 18 years or older, in their third trimester of pregnancy (GA ≥ 28 weeks), fluent in Norwegian, residing in the western regions of Norway, and capable of providing informed consent and completing the interview. Eligible participants expressing interest in the study provided their contact details on a dedicated recruitment site hosted by Nettskjema. Following submission, EK-D reached out to provide verbal information about the study. Women agreeing to participate in the study, received written information about study details and consent forms via Nettskjema. Once consent was obtained, participants completed a digital background questionnaire including age, parity, educational level, previous PGP, GA during pregnancy, through the same platform. The questionnaire also contained the pelvic girdle questionnaire (PGQ), which is a patient-reported outcome measure to specifically assess activity limitations and symptoms in women presenting with PGP during pregnancy and postpartum. The PGQ scores are summarised and converted to percentage scores for easier interpretation, ranging from 0%, indicating no problems, to 100%, indicating greater disability or more severe symptoms [29]. Interviews were recorded using Nettskjema’s dictaphone app. We aimed to recruit a purposive sample of 20 women. Saturation was assessed using a thematic saturation approach, whereby data collection continued until no new themes or meaningful variations in the data were identified. Analysis was conducted iteratively alongside data collection. After 18 interviews, additional themes yielded no new themes and served to reinforce the existing thematic framework. Therefore, it was assessed that further data collection was unlikely to substantially change the thematic structure.

### Datacollection

The interview guide was developed by EK-D and AMG based on existing literature and clinical experience. Once the draft was complete, user involvement feedback was sought from women in the association *Landsforeningen for kvinnelig bekkenleddshelse* (LKB) and the researchers of the project group PGP-NOR. This feedback resulted in minor revisions to ensure clarity and alignment with the research aim. The guide consisted of open-ended, neutrally worded questions in Norwegian, with follow-up prompts initiated by EK-D during the interviews. A pilot interview was conducted with a woman that met all the inclusion criteria and provided a signed consent. The pilot interview was used to assess the clarity, relevance, and flow of the interview questions, as well as the participants’ understanding of the topics. As no issues were identified, no changes were made to the interview guide, and the pilot interview was included in the analysis. After completing the fifth interview, EK-D, AMG, and CFR met to review the interviews and assess whether changes to the interview guide were necessary. No new questions were added following this discussion.

Data collection took place from December 4, 2023, to May 1, 2024, through semi-structured in-depth interviews, a method that allows for dynamic exploration by addressing and following up on interesting or unexpected responses, in contrast to more rigid structured interviews [30]. EK-D had no knowledge of the participants prior to the interviews. Throughout the interviews, participants were encouraged to provide more detailed and thoughtful responses by asking probing questions. A summary of the interview guide can be found in Appendix 1. All interviews were performed by EK-D except for one, in which AMG assisted with interviewing. To minimise the burden on participants, interviews were scheduled at convenient times for the participants and offered at two locations to reduce travel time: Stavanger University Hospital and Klinikk for Alle Bryne. For participants unable to travel, an online option was made available via Microsoft Teams. Two interviews were conducted via Microsoft Teams due to logistical constraints. The online interviews did not differ from the in-person interviews in terms of duration or depth of responses. The interviews were all conducted in Norwegian and lasted between 16 and 56 min, with a mean duration of 30 min. The mean coded segments for interviews yielded 106.5, with a standard deviation (SD) of 46.2. All participants provided verbal and digitally signed consent. EK-D maintained a reflective journal throughout the process, documenting critical reflections, personal assumptions, and observations. All the women gave permission for their interviews to be recorded. The interviews were automatically transcribed using Nettskjema’s integration with Whisper by OpenAI. However, due to dialectal variations among the participants, the transcriptions required manual correction by EK-D to ensure accuracy and verbatim representation.

### Qualitative analysis

Considering the extensive information gathered during the in-depth individual interviews, the data was divided into two parts. This article focuses on the first part, which explores the women’s personal experiences of living with PPGP, including its impact on their quality of life and family dynamics. A qualitative content analysis (QCA) according to [31, 32] was used to stepwise analyse the transcriptions. The selected analytical approach effectively facilitates a thorough exploration of both obvious and underlying differences and similarities in the data [31, 32]. The MAXQDA ^®^ software program [33] was used to aid in the procedure of coding and the development of categories. Initially, the transcriptions were read repeatedly to identify areas of content interest. The content was further discussed between EK-D, CB and AMG. Secondly, sentences and phrases relevant to the study’s objectives were extracted, condensed, and rephrased to preserve their original meaning. These condensed units were then labelled with codes. Any discrepancies in coding were discussed iteratively among EK-D, CB, and AMG in consensus meetings until agreement was reached. The codes were compared to identify similarities and differences and were organised into clusters based on shared content. These clusters were subsequently grouped into categories, reflecting the manifest aspect of the analysis. Emerging findings were shared with all co-authors for further feedback and validation. In the final stage, themes capturing the latent meanings across the data were identified. The authors’ diverse professional backgrounds, including three chiropractors, two of whom hold PhDs, and a midwife/public health nurse with a PhD that provided extensive expertise in qualitative research. The combination of their clinical experience strengthened the confirmability and rigor of the analysis. Relevant quotes supporting the findings are provided in the results section.

The Consolidated Criteria for Reporting Qualitative Research (COREQ) checklist [34] was followed.

## Results

Eighteen women, with a mean age of 32 years, were interviewed between GA 29 and 39. All the women were married or cohabitating, they were all gainfully employed full/part-time, and most women reported that they had been or were on sick leave, in part or full. All women reported a singleton pregnancy, and 67% were multiparous. Most women did not have physically demanding jobs, however, 67% (*n* = 12) of the women reported being physically active. A complete demographic overview is provided in Table 1.

**Table 1:** Summary of sociodemographic, health and pregnancy in 18 women with PPGP

Variable	*N*	Mean; SD^a^
Age		31.6; 4.5
Highest level of education		
High school	2	
Vocational certificate	1	
Bachelor	6	
Masters	9	
Previous pregnancies		
No previous children	6	
1	8	
2	3	
3	1	
PPGP in previous pregnancy/ies		
Yes	8	
No	10	
Gestational age during interview		32; 3
PGQ score percentage		62,2; 19

*SD* standard deviation, *PPGP* pregnancy-related pelvic girdle pain, *PGQ* pelvic girdle questionnaire

### Overarching theme

Through the analysis, a central theme that emerged was: *This*,* too*,* shall pass.* This theme is rooted in participants’ narratives portraying PPGP as an expected, albeit unwelcome, aspect of pregnancy, something to be endured until childbirth. While many expressed hope that the pain would subside immediately postpartum, others held no such illusions, recognising that recovery might take time. Still, an overarching expectation persisted: that the pain would eventually pass, and life would return to normal. Across accounts, women voiced a strong desire to adapt and maintain a balance during pregnancy, both to cope with daily challenges and to minimise the risk of persistent PGP. The theme captures a fundamental tension between endurance and self-preservation. Participants described the emotional and physical strain of navigating PPGP symptoms while striving to uphold their roles as mothers, partners, and professionals. The belief that “This, too, shall pass” became a recurring narrative, both a coping mechanism and a framework for interpreting their experience, shaping how they managed their condition and made decisions throughout pregnancy.

Participants’ narratives illustrate the profound impact on nearly every aspect of their daily lives. Most women reported a substantial decline in both physical and emotional functioning. Routine activities such as walking, cleaning, or simply sitting down with their children became increasingly difficult. In addition, women were forced to carefully balance their activity levels, incorporating frequent breaks and position changes to avoid exacerbating their pain. While the majority expressed a desire to remain at work despite symptoms, they also acknowledged the challenge of managing full-time work alongside childcare responsibilities. Workplace accommodations, when attempted, were largely ineffective, often resulting in partial or complete sick leave Table 2.

**Table 2:** Example of the analysis

Meaning unit	Condensed meaning unit﻿	Code	Category	Subtheme
"In the beginning he would copy me. When he saw may back hurt, his back also hurt. I don't know if he understands, but he has accepted it. That is hurtful."	He would copy me at first. When he saw my back hurt, he would copy me. He might not understand, but he has accepted it."	The children have accepted mum's limitation to participate	Children's acceptance	Perceived understanding and support from others

Pain was consistently present both day and night, with minimal relief, often resulting in disrupted sleep and accumulated exhaustion. Despite these challenges, support from partners was universally highlighted as a crucial buffer, and most participants felt validated and encouraged when discussing PPGP with others. The depth and complexity of these experiences are further illustrated through participants’ quotations, grounding each of the four sub-themes in the lived realities of the women themselves (Table 3).

**Table 3:** Illustrative overview of the findings

Overarching theme	Sub-theme	Category
This, too, shall pass	Internal conflicts affecting self-image	Adapting to and balancing physical limitations
		Self-blame and feelings of inadequacy
	Forced to deal with an unwanted situation	Unwelcome new role and adjusting to a new normal
		Ambiguity towards sick leave
	Never catching a breack from pain	Impact on daily activities
		Limited moments of relief
		Sleep disturbance due to pain
	Perceived support from others	Partner as support/er
		Family and peer-support system

##  Internal conflicts affecting self-image

Participants reported experiencing a wide range of emotions in response to PPGP, including frustration, annoyance, and a deep sense of unfairness. The severity of these feelings varied among individuals. Many expressed sadness over the loss of physical functioning, highlighting how the pain negatively impacted their daily lives. The inability to perform routine tasks contributed to feelings of inadequacy and threatened participants’ sense of identity as capable mothers and partners. Furthermore, the pain diminished their ability to fully enjoy the anticipation of their baby’s arrival. This sub-theme comprises two categories presented below: “Adapting to and balancing physical limitations” and “Self-blame and feelings of inadequacy”.

### Adapting to and balancing physical limitations

Participants expressed challenges in adapting to the physical limitations imposed by PPGP, with many struggling to accept the loss of their independence and necessity to alter their daily routines. Some women found it hard to slow down and delegate tasks, feeling a strong internal urge to stay active despite discomfort.

*I used to exercise actively. I did heavy strength training*,* which was my daily routine. Then suddenly I had to spend half an hour just sitting on a ball to work on mobility in my pelvis and hips. That’s quite a big leap from what I’m used to. It was also a major mental shift. (Participant 7)*

Others expressed a feeling of frustration and helplessness when confronted with even simple tasks, such as walking short distances, which had become overwhelming. Balancing self-care during pregnancy with the demands of childcare further complicated their ability to prioritise their well-being.

*If I manage to strike a balance and find that middle ground*,* I can sense when I need to take a break. I’ll lie down to rest and then have the energy to tidy up and get things done. Lie down and relax. Other times*,* I keep pushing through*,* and then everything just locks up. Then you spend the whole evening just looking forward to going to bed*,* because that’s when you can finally relax. (Participant 12)*

Many participants observed a disconnect between their physical limitations and their desire to maintain previous activity levels, leading to feelings of demotivation and frustration. Ultimately, reconciling the need to seek support while accepting their limitations was a significant and ongoing challenge for several women.

*One might lose motivation when you’re not able to do what you really want. The mind wants to*,* but your body is saying no. (Participant 12)*

### Self-blame and feelings of inadequacy

The emotional toll of PPGP was profound, with participants struggling to reconcile their physical limitations with their desire to be active and engaged mothers. Many felt sidelined in their children’s lives, experiencing frustration and sadness at their inability to participate fully in everyday activities such as carrying, playing, and engaging in family routines. This loss of involvement contributed to a deep sense of disappointment and self-doubt.

*It is hard not to be able to participate in the kids’ activities. So*,* we try and adjust so I can join in on some things. (Participant 14)*

While some women adapted by finding alternative ways to stay involved with their families, others found it difficult to accept their reduced capabilities, feeling as though they were becoming a ‘lesser’ version of themselves. The constant need to balance physical limitations with the demands of motherhood required ongoing adjustments and compromises, further adding to their emotional burden.

*I understand that experiencing pelvic girdle pain*,* feeling nauseous and getting little sleep also affects the pelvis*,* and has a massive [negative] impact on me*,* causing me to become a worse version of myself as a mother*,* first and foremost. (Participant 9)*

Participants expressed a strong desire to remain present with their children to the best of their ability despite their physical limitations. When unable to engage physically, they sought to compensate by being more mentally and emotionally available, such as minimising distractions and encouraging their children to spend time with them in ways that required less physical effort.

*I try to be present as much as I can*,* in the ways that I can. I’ve become more conscious about putting my phone away to be mentally present. He’s [the child] understood for a while now that I won’t go along when they go to the playground*,* so he doesn’t ask anymore. He accepts that I lie on the sofa and participate in the play from there. He accepts that his dad must do more of those things now than he did before. (Participant 2)*

Some women expressed frustration about the unpredictability of pregnancy experiences, believing it was unfair that some had uncomplicated pregnancies while they struggled with pain. Doubts about whether others truly understood their condition added to their emotional burden, as they feared being perceived as exaggerating their discomfort. The constant pain and lack of comfortable positions drained them of energy.

*Of course*,* it limits me*,* but at the same time*,* it could have been much worse. I can still move around. What I find the hardest now is that*,* like everyone*,* I want to be the best version of myself as a mother. (Participant 9)*

For many women, regardless of whether they had children previously, the most overwhelming feeling about experiencing PPGP was a deep sense of unfairness. It took away from the joy of pregnancy, leaving them struggling with frustration and unanswered questions. The injustice some women felt was profound. The lack of a clear cause made it even harder for them to accept why this had happened to them and not to others. The uncertainty of not knowing how, why, or what triggered their PPGP was, for some, the most challenging aspect to come to terms with. For numerous women, the only way to cope was to endure, holding onto the hope that once their baby arrived, the pain would finally subside.

*Why do I get pelvic girdle pain? Why do I get pelvic instability? Why am I in pain while others aren’t? What am I doing wrong? What should I be doing differently? Is it because something is tight? Should I be stretching? If I had done strength training? Why do I get this… I’m missing a cause*,* something to help make sense of it*,* in a way. (Participant 18)*

The condition is not only physically demanding but also emotionally exhausting, forcing women to make major lifestyle adjustments. While most feel their daily lives have changed beyond recognition, a few manage to maintain their routines despite the persistent pain, though it remains a constant factor in their day-to-day experiences.

*First and foremost*,* it’s life-limiting. You have to alter your lifestyle. You can’t do the things you used to do. It feels like you’re falling a bit outside the community. (Participant 8)*

### Forced to deal with an unwanted situation

Many women were unprepared for the impact of PPGP, which disrupted their expectations of pregnancy and life after childbirth. Many worried whether the pain would persist postpartum, with some hoping for immediate relief while others anticipated a longer recovery. A common frustration was the inability to remain as active as they had hoped, particularly those with older children. However, expectant mothers with older children found ways to adapt, remaining engaged in their children’s lives despite their limitations. Workplace experiences were equally complex. Many women, especially those in physically demanding jobs, struggled to obtain necessary accommodations, often leading to reduced hours or complete sick leave. Some felt guilt or faced scepticism from colleagues, which prompted them to downplay their condition. Those who continued working found it increasingly difficult to balance their responsibilities, and stepping back from their professional roles represented a significant emotional adjustment. Ultimately, some opted to leave work entirely, prioritising their well-being and their ability to care for their families. This sub-theme consists of two categories: “Unwelcome new role and adjusting to a new normal”, and “Ambiguity towards sick leave and workplace adaptation.”

### Unwelcome new role and adjusting to a new normal

Participants expressed significant concerns about the potential persistence of their pain and struggles beyond childbirth, emphasising the emotional burden of adapting to a new normal. While some hoped for gradual relief, they also worried that their difficulties would not simply disappear after childbirth. Others acknowledged the need for ongoing adjustments, accepting that their challenges might extend well into motherhood. Pregnancy was frequently characterised as something to endure, with a focus on persevering through physical discomfort until after childbirth. Some women, particularly those who experienced severe PPGP, felt reluctant to undergo another pregnancy.

*Now it’s truly just about gritting my teeth and getting through it. I’m trying to keep my body moving so that I come out of it as well as possible after the baby is born*. *(Participant 7)*

Women with older children described how their children gradually adapted to these changes, learning to accept that their mothers could no longer participate in activities as they once had due to pain. However, for some, this adjustment was emotionally challenging, as they observed their children either mirroring their discomfort or struggling to understand the limitations imposed by their condition.

*At first*,* he [the child] copied me. When he saw that I was in pain*,* he said his back hurt too. I don’t know if he really understands*,* but he has accepted it. That part feels a bit painful. (Participant 2)*

Mothers of younger children faced even greater obstacles as their little ones struggled to understand and accept these new constraints. Many children became more emotionally demanding, leading to frustration and distress, particularly when other caregivers stepped in to assist. Despite these challenges, mothers remained determined to stay involved in their children’s lives, finding creative ways to engage from a distance, whether by reading at bedtime, participating in play from the sidelines, or simply being present in a more passive role.

*I am present*,* it’s not like I’m bedridden. I’m downstairs in the living room*,* and I have been present on the side. However*,* I’m still on the sideline. (Participant 11)*

When asked how others might perceive PPGP, a few women mentioned that it might be understood as exaggerated to some individuals. However, they themselves came to truly understand it only after personally experiencing it.

*I think it’s easy for people to believe it’s a bit exaggerated. That’s what I thought myself before I got pregnant. It can’t be that bad*,* I thought*,* but now I understand. (Participant 6)*

Some women reported finding comfort in reminding themselves that, despite the challenges of PPGP, it could be worse, at least it wasn’t affecting their baby like gestational diabetes or preeclampsia.

*When we decided to have another child*,* I knew it was going to be like this because I had problems during my first pregnancy. […] I feel fortunate because I’ve pelvic girdle pain*,* but no complications with the baby. (Participant 11)*

### Ambiguity towards sick leave

Participants reported mixed experiences with sick leave and workplace accommodations, with many facing challenges balancing work and physical discomfort. Those in physically demanding jobs often struggled to receive adequate adjustments, resulting in part- or full-time sick leave. This decision was not always easy, as some felt guilt when comparing themselves to colleagues who worked through pregnancy. In contrast, others encountered negative reactions when disclosing their condition, leading them to keep it private for as long as possible.

*At work*,* that’s where I feel it the most. I really want to make it to maternity leave. I think I’ll manage with the way I’ve planned things now. (Participant 12)*

For many, continuing to work without significant accommodations became impossible due to pain. Some found that reducing their workload helped manage symptoms, enabling them to remain engaged at home as mothers. However, even with adjustments, balancing work responsibilities alongside pregnancy-related pain was exhausting. Those who worked until maternity leave described the increasing difficulty of managing their condition. Beyond the physical strain, participants highlighted the emotional toll of stepping back from their professional roles. Many had never been on sick leave before and found the transition from being professionally active to reducing or ceasing work entirely a significant adjustment, often accompanied by frustration and a sense of loss.

*I really enjoy working; I’ve never been on sick leave before. Suddenly*,* I’m not going to work. It’s a big transition. (Participant 4)*

The nature of participants’ work also influenced their experiences. Women in physically demanding jobs often had to take more frequent breaks or make significant adjustments to their tasks, while office workers encountered challenges related to prolonged sitting or limited opportunities for movement. Ultimately, some participants chose to cease working entirely to conserve energy for their families, prioritising their well-being and ability to care for their children over professional commitments.

*I have sacrificed work so that I can be a mother in the afternoon when she returns home from preschool. (Participant 11)*

### Never catching a break from pain

Women experiencing PPGP described the pain as distinct from common discomforts like back pain, often characterising it as life-altering. This intense pain forced many women to significantly adjust their daily routines. Simple tasks, such as climbing stairs in their own homes, became so daunting that they would avoid them altogether. To manage their day, women had to carefully plan when to engage in activities and when to rest. Maintaining a balance between everyday life and managing their condition proved challenging for most, as it required accepting a reduced level of physical activity compared to what they were accustomed to or hoped for during pregnancy. To alleviate the pain, frequent changes in position became essential. A common pattern observed was the pain intensifying throughout the day, peaking in the evening, and leading to disrupted, painful sleep. The poor quality of sleep often necessitated daytime naps for some women to function, though for others, sleep provided a temporary “reset” that allowed them to face the next day. This sub-theme comprises three categories presented below: “Impact on daily activities”, “Limited moments of relief” and “Sleep disturbances due to pain”.

### Impact on daily activities

For all the women, the impact of PPGP on their daily lives was profound and life-altering. They were forced to modify their routines, including emptying the dishwasher, vacuuming, cooking and taking their children to nursery by incorporating more frequent breaks and rest periods. Transitioning from a life free of movement concerns to one where every action had to be carefully planned was overwhelming, and many struggled to accept this significant shift. For some women, moving around the house was exceptionally challenging, particularly when navigating stairs. While housework became difficult for many, it was one of the few tasks they felt capable of doing in short bursts to contribute to household chores. However, these activities had to be carefully scheduled throughout the day or week and were often completed to a lower standard than they were accustomed to. To alleviate pain, women commonly alternated between sitting, lying, standing, and walking, as remaining in any one position for too long tended to worsen their discomfort. Most women also reported significant pain when getting in and out of the car, with the twisting motion of swivelling into a seated position exacerbating the pain.

*Right now*,* I feel like I have to take more breaks and rest much more than I’m used to. The more I move*,* the more it hurts. […] It’s bothersome and painful all the time. (Participant 4)*

Reducing work hours provided women, particularly those expecting their second or third child, with an opportunity to recover between work and childcare responsibilities, allowing them a better chance to remain active. However, when their children were at home, taking necessary breaks became more difficult, and certain tasks, such as carrying or comforting their children, were unavoidable. Many women accepted that these actions would lead to an increase in pain, knowing they would experience the consequences later.

*I’m not working full-time at the moment. I’m working 40%*,* and honestly*,* that’s enough. I struggle a lot with guilt. But you know*,* there’s drop-off and pick-up at preschool*,* and everything at home still needs to be done*,* whether you’re pregnant or not. (Participant 9)*

A common theme among women was the fear of experiencing persistent pain after delivery. While some were advised by healthcare providers or informed by friends and family to take it easy and not push themselves, for many, it was an insight they arrived at intuitively on their own. For some of the women whose mothers are still suffering from the consequences of PPGP, preventing them from playing with their grandchildren or performing physical activities they could before their pregnancies, the warnings passed down were firm: listen to their bodies and do not push through.

*My mom told me she had it [PPGP]when she was pregnant with me. […] She told me to take it seriously. That I shouldn’t even think about going for a jog*,* because that’s not good. (Participant 4)*

### Limited moments of relief

Many women expressed a desire to remain active, but any physical activity exacerbated their pain. A recurring theme among them was the struggle to find balance, with many resonating deeply with the challenge of navigating this issue. The most difficult aspect was identifying a level of activity that wouldn’t intensify the pain. For some women, the pain intensified with activity. Most women reported that the pain progressively worsened throughout the day, often peaking at night. Another common experience was the unexpected severity of PPGP, leaving many unprepared for the extent to which it would limit their daily activities.

*It worsens in the afternoon and evening*,* especially if I’ve had a busy day. I also find it difficult because I want to be active*,* move*,* and exercise as much as possible. Finding that balance has been challenging. (Participant 4)*

Some women reported not feeling pain during or immediately after an activity, but the effects would manifest the following day when they woke up. However, a few women chose to push through, engaging in activities despite knowing it would exacerbate their pain. Few women reported that the pain affected their daily lives by making them perform tasks more slowly, but not to the extent that it hindered their ability to carry out the tasks they desired.

*I have been in pain*,* but it hasn´t hindered me in doing what I´ve wanted to do. I have had to pause running*,* but I am still able to participate in activities. (Participant 3)*

### Sleep disturbances due to pain

All women reported disrupted sleep, not only as a direct result of PPGP, but because movement during sleep, as well as falling and staying asleep, had become increasingly difficult. Many women described a persistent ache that made turning over difficult, with some needing assistance from their husbands to get out of bed or change positions. For some, the prospect of catching up on sleep after sending their children to preschool or school was essential for maintaining their motivation to endure the pregnancy. While a few women reported that sleep provided a temporary sense of relief, leaving them feeling fine after a night’s sleep, only for the pain to worsen throughout the day, the majority described minimal relief from pain at any point during the day or night.

*What’s new in the past month is that I’m having trouble falling back asleep. […] I’ve been lying awake for an hour*,* and during that time*,* you become even more aware of the pain. […] I mean*,* I do sleep. I’m on sick leave*,* so I can recover during the day. (Participant 1)*

A few women reported that their sleep during this pregnancy was notably different from that in previous ones. Disrupted sleep due to a toddler’s needs compounded the challenge, while the sensation of “locked” joints, which restricted movement, made it particularly difficult to get out of bed, even for basic tasks such as using the bathroom.

*Last time*,* I struggled with sleep because of the pregnancy. But now I’m also struggling with sleep because I have a daughter who sleeps very poorly at night. (Participant 9)*

### Perceived support from others

Perceived support from othersSupport was often described by the women as the most essential factor in managing the challenges of PPGP. They frequently expressed that their partner was their primary source of support, whether it involved helping with household chores, childcare, or providing emotional reassurance. Despite this, many women found it difficult to relinquish control over tasks they had previously handled themselves. This dynamic became especially evident when children seemed to struggle with understanding why their mother couldn’t participate in activities despite being physically present. For some women, help from extended family members was crucial to their ability to remain active in parenting. Validation and support from others helped the women feel understood and less isolated in their struggles. Below, this sub-theme is broken down into two categories: “Partner as support(er)” and “Family and Peer support system”.

### Partner as support/er

Many women described their partners as highly supportive, taking on extra responsibilities at home to help manage the challenges of pregnancy. Some partners naturally assumed a larger role handling household chores, childcare, and daily routines, while others needed prompting to understand and respond to their partner’s needs. Women’s primary concern was ensuring their children received the care they needed, leading them to place greater importance on their partners’ involvement in parenting rather than other domestic tasks. Consequently, many deferred parenting duties to their partners out of necessity rather than preference. Several women felt fortunate to have such engaged partners, recognising that this was not the case for everyone.

*He’s [husband] made a huge effort. He’s really good that way. She [the child] is quite attached to her dad. It’s exhausting for him*,* too. He’s had to drop her off and pick her up from preschool and he’s the one who puts her to bed as well. (Participant 11)*

For many, their partner’s increased involvement provided much-needed relief, particularly in managing cooking, cleaning, and bedtime routines. Some coped with these limitations through humour, joking that their partner should take on a potential subsequent pregnancy. However, this shift also introduced emotional and relational challenges. Ultimately, while partner support was vital in managing daily life, it also created underlying tensions between the need for assistance and the desire to maintain independence, leaving women balancing feelings of gratitude, frustration, and a sense of loss of control.

*Because when you’re in constant pain*,* it’s exhausting to deal with people. It takes so much of your focus. You end up being that mum who sits there while your partner does most of it. And at the same time*,* it’s just a fraction of everything going on inside the house that’s visible from the outside. (Participant 8)*

### Family and peer support system

Participants also emphasised the importance of connecting with others who had experienced PPGP, as they provided genuine understanding and empathy that others often could not. Friends and colleagues with a history of PPGP were regarded as particularly supportive, offering validation and acknowledging the severity of the condition.

*I feel that I’ve been well understood by my doctor*,* midwife*,* colleagues*,* boss*,* as well as friends and family. Whether they fully understand*,* I don’t know*,* but they believe what I say. (Participant 2)*

While some women felt well-supported by healthcare providers, colleagues, and supervisors who accepted their accounts of pain and limitations, frustration arose when individuals without firsthand experience claimed to relate but failed to comprehend the true impact of the condition. There were a few women who appreciated that others understood, but did not feel that it aided the acceptance of the situation and offered little relief for the suffering woman.

*It kind of depends on who you talk to. If you talk to people who’ve had it themselves*,* there’s a completely different level of understanding. Those who haven’t been pregnant*,* or haven’t had it [PPGP]*,* see it as just another typical pregnancy complaint. (Participant 12)*

Some women refrained from expressing their discomfort, fearing that doing so would lead others to perceive them as weak or complaining. They preferred to maintain a sense of privacy, particularly in work-related situations by not elaborating further on the pain. Faced with colleagues who did not understand, they often felt compelled to explain and justify themselves to avoid negative judgment.

*Especially at work*,* I get the sense that many people think pregnancy isn’t an illness*,* and maybe they haven’t experienced it [PPGP] themselves*,* or had a very easy pregnancy*,* you end up feeling like you must explain and justify yourself. (Participant 9)*

For women with children, the support provided by extended family was crucial to managing childcare responsibilities. For some, it allowed them to take their children to the playground, knowing that others could assist in caring for the children if necessary. This support was particularly significant, as it enabled the women to have meaningful interactions with their children, ensuring they could still share experiences together despite their physical limitations.

*I also have parents who make it easy for me to participate in those kinds of activities with them [the children]. We find our own way. […].What matters is that we get out of the house and have an experience. There’s a strong focus on creating experiences and doing things together. (Participant 8)*

## Discussion

This study aimed to explore experiences of Norwegian women living with of PPGP, with particular emphasis on symptoms, coping, strategies and the impact of health-related quality of life (HRQoL) and family dynamics. The findings reveal that PPGP significantly impairs women’s physical functioning, emotional well-being, and capacity to manage daily activities. Participants consistently reported feelings of frustration and a sense of injustice linked to the loss of independence and the ongoing need to navigate a careful balance between activity, rest, and parental caregiving duties. Coping often involved practical adaptations and a considerable reliance on support from partners and families. Furthermore, many women described a disruption to their sense of identity and experience of stigmatisation related to taking sick leave due to their symptoms. These findings illuminate the substantial and multifaceted impact of PPGP on women’s everyday lives, workforce participation, HRQoL and family relationships.

Consistent with previous research, our findings reinforce that PPGP is experienced as both physically disabling and emotionally demanding [13, 35–38]. The cumulative impact of persistent pain, functional limitations, and disruptions across all domains of daily life may frequently contribute to psychological distress, including low mood and symptoms of depression [13]. Common coping strategies may involve carefully planning activities, frequently changing positions, and weighing whether the perceived benefit of an activity justifies the anticipated physical consequence [37, 39]. Participants in this study often described a profound loss of independence. Routine tasks such as walking, bending or carrying laundry up a flight of stairs frequently required modification or external support, further contributing to social withdrawal and isolation. The inability to complete household tasks, such as cleaning, vacuuming, or doing laundry, elicited strong emotional reactions, including frustration, anger and sadness. While a minority of women managed to maintain certain routines, most reported significant disruptions to daily life. Beyond these physical challenges, many participants described PPGP as an attack on their sense of identity, often expressing feelings of being a diminished version of themselves. To cope, some women avoided household tasks entirely, while others completed them in brief intervals, adapting their routines to fluctuating pain levels.

A persistent conflict emerged between the need to remain active and the necessity of rest, as women navigated the unpredictable course of their symptoms. Pain severity varied considerably, from mild discomfort to debilitating distress, and participants were acutely aware that overexertion often triggered symptom exacerbation. This created a continual dilemma; to remain functional at the risk of intensifying symptoms, a pattern also observed among women with persistent PPGP postpartum [37]. The invisible nature of PPGP may further compound its psychological toll, where women often question the legitimacy of their suffering when symptoms are not outwardly visible or acknowledged by others, leading to diminished self-trust and hesitancy to seek help and support [13, 39]. These experiences are consistent with broader literature on invisible chronic conditions [40, 41] where the lack of visible markers challenges individuals’ confidence in their bodily perceptions and complicates interactions with healthcare providers and social networks [42]. Among first-time mothers, especially, unfamiliar physical sensations and the absence of prior reference points may increase uncertainty and contribute to maladaptive illness behaviour, characterised by heightened concern, avoidance of activity, or excessive help-seeking [43]. In this context, receiving validation and a clear explanation of PPGP from healthcare professionals can be a source of reassurance and legitimacy for many women [43].

A recurring pattern among the participants was the worsening of pain throughout the day, often peaking in the evening and severely disrupting sleep. This is consistent with research linking musculoskeletal pain to increased risk of sleep disturbances, particularly among individuals with low back pain [44]. Moreover, studies suggest that up to 70% of women with PPGP experience poor sleep quality, which may contribute to pain chronicity and increase the risk of persistent symptoms postpartum [37, 39, 45]. While some women in this study described sleep as a temporary reprieve from pain, others reported experiencing disrupted and insufficient rest, often requiring daytime naps to compensate for sleep deficits.

### Motherhood and the emotional burden of role restriction

In the context of motherhood, these physical and emotional challenges manifested as role restriction and concerns about maternal adequacy. While previous research suggests that pain may compromise women’s ability to meet caregiving demands [11, 39, 46], participants in this study described adaptive strategies to remain emotionally present for their children despite limited mobility. Nevertheless, being unable to participate fully in caregiving elicited guilt, self-doubt, and emotional strain, particularly among mothers of younger children, echoing prior findings on the psychological impact of PPGP on maternal identity [11, 13]. These feelings may reflect the high expectations women place on themselves in their caregiving roles, potentially contributing to the higher prevalence and burden of PPGP among multiparous women compared to first-time mothers [47].

Over time, families adjusted to the evolving demand of living with PPGP, yet many women continued to experience a persistent sense of reduced capability, often accompanied by feelings of grief and guilt. The impact of persistent pain also extended into close relationships, where participants described increased emotional reactivity and reduced patience as common consequences of physical and emotional exhaustion. Although participants in the present study did not report increased conflict with their partners, previous research has identified increased relationship tensions among some women, attributing these strains to the ongoing emotional burden of living with PPGP [11]. Partner support was widely regarded as crucial in managing daily life, although it was not without its complexities. Many partners assumed greater responsibility for childcare and household tasks, enabling women to conserve energy and manage their symptoms. However, this redistribution of roles occasionally resulted in emotional strain, particularly as women struggled with the loss of independence and control over routines they had previously managed with ease. These experiences resonate with earlier findings that highlight emotional challenges associated with role redefinition and dependence within the family dynamic [11, 13].

Notably, participants described partner support as emerging from necessity rather than preference. Consistent with previous studies, partner support was essential in managing daily life with PPGP, although often emerging from necessity than preference [10, 11]. While redistribution of household and caregiving roles enabled women to conserve energy, it also reinforced feelings of dependence and loss of control previously described in relation to identity disruption. Although this shift was sometimes accompanied by frustration, participants frequently described their relationship as a collaborative effort, emphasising the importance of working together to manage daily life. Despite the central role partners often play in managing PPGP, the broader literature offers limited insight into their experiences and perspectives. When examined, evidence suggest that partners may experience considerable emotional strain, particularly when attempting to balance employment, caregiving responsibilities and provision of emotional support [48]. Several male partners reported feeling deeply connected to their partner’s well-being, noting that the severity and persistence of PPGP symptoms had a direct impact on their own mental health [48]. When available, extended family members played a meaningful role in supporting family functioning and easing the daily burden of PPGP. Assistance from parents and in-laws was particularly appreciated, enabling participants to remain engaged in daily routines and helping maintain a sense of normalcy in family life despite physical limitations. This finding is consistent with previous research highlighting the importance of informal support networks in mitigating the impact of PPGP [11, 17].

First-time mothers often expressed uncertainty regarding the course of recovery, lacking prior reference points to contextualise their symptoms. In contrast, participants with previous experience of PPGP reported a more optimistic outlook, drawing on their earlier experiences to inform their expectations and coping strategies, congruent with prior research [36]. Similarly, women with a family history of PPGP, such as mothers, aunts and mother-in-law, also described being more attuned to bodily signals and more inclined to “listen to their bodies”, which influenced their coping strategies. Nevertheless, even among participants with greater understanding or prior knowledge, this awareness did not necessarily translate into greater emotional acceptance.

### Workplace challenges and identity loss

In Norway, there is a strong societal expectation for women to remain in the workforce throughout pregnancy. This is particularly evident when symptoms are viewed as “normal pregnancy complaints”, such as sleep disturbances, back pain, and fatigue [49]. However, the line between typical pregnancy experiences and what qualifies as a disabling condition is often unclear. Many women suffering from PPGP find their realities blatantly contrast with these expectations, experiencing significant physical limitations that lead to feelings of disconnection from their bodies. They often express a profound sense of being trapped in a body that feels unfamiliar and unreliable [50]. Workplace stigma can exacerbate these difficulties, with women feeling compelled to hide the severity of their conditions for fear of being judged, not believed, or being seen as less capable [11]. The prevailing social narratives around “normal pregnancy” significantly influence both how women with PPGP view themselves and how they are viewed by others [38]. While PPGP is being increasingly recognised in clinical settings today, many study participants reported feeling overwhelmed by the physical demands of their jobs, all while grappling with the conflicting notion that, despite pregnancy not being classified as an illness, their condition still justified taking sick leave.

It has been suggested that PPGP can no longer be viewed as a normal part of pregnancy, given its significant physical and psychological impact on affected women [51]. PPGP has been identified as the second most common cause of sick leave during pregnancy [52], with studies indicating a strong link between PPGP and higher sick leave rates in late pregnancy, particularly in high-employment countries like Norway [47]. Furthermore, requests for extended sick leave are often met with scepticism, leaving many women concerned about being perceived as exaggerating their symptoms [49]. This reflects the broader, ongoing challenge of legitimising pregnancy-related conditions as valid reasons for work absence [49]. In Norway, although pregnant women are legally entitled to workplace accommodations, those employed in physically demanding occupations, such as shift work or manual labour, often face significant challenges accessing suitable modifications. As a result, many are left with no alternative but to take partial or full sick leave [53]. However, the decision to take sick leave has also been shown to be motivated by a desire not to overburden colleagues, reflecting the tension between personal limitations and professional responsibility [50]. For several participants in this study, taking sick leave was a complex emotional experience. Work was described as a central component of their identity, and the inability to perform their professional duties elicited feelings of frustration, loss, and a diminished sense of self-worth.

The concept of the “double shift”, balancing paid employment with domestic responsibilities, has been identified as a significant factor contributing to burnout and the eventual withdrawal from the workforce, particularly among women [53]. For those experiencing PPGP, this dual burden becomes especially overwhelming. Many women in our study reported continuing to work despite substantial physical discomfort, all while managing household duties and caregiving roles. This cumulative strain not only worsened their physical symptoms but also led to emotional exhaustion, heightened stress, and a pervasive sense of inadequacy. The struggle to meet expectations in both spheres can trigger feelings of guilt and frustration, and for some women, it may ultimately result in either temporary or permanent withdrawal from the workforce. Therefore, there is a critical need to enhance societal awareness of PPGP and strengthen support systems. Without adequate recognition and workplace accommodations, there is a risk for extended disability leave, which carries significant costs for both the individual and society through reduced workforce participation and increased economic costs [21].

### Methodological considerations/strength and limitations

Recruitment sites were selected to include both urban and rural healthcare settings, thereby ensuring a geographically diverse and representative sample. Interviews were conducted in a various environments, including clinical offices, general office spaces, and via online platforms. While this variation may have influenced how participants responded, it also reflects the flexibility required to accommodate women at different stages and contexts of pregnancy. Another strength of the study is its inclusion of both first-time mothers and women with previous children, providing a broader perspective on how PPGP affects family dynamics, including parenting responsibilities and partner relationships.

Although only one interviewer conducted the interviews, multiple authors reviewed transcripts prior to analysis, and the process of coding and theme development was conducted collaboratively, enhancing the credibility and trustworthiness of the findings. Participants also completed the PGQ, which provided insight into their functional status and indicated that they suffered from PPGP, not LBP. The wide range of scores suggest a heterogeneous presentation of PGP in the group, reflecting the diverse impact of the condition on women’s daily activities and quality of life.

This study required participants to be fluent in Norwegian, which may have excluded women from ethnic minority backgrounds who might hold different perspectives on partner involvement, pain communication, and access to care [54]. Additionally, two-thirds of the participants held a university degree, whereas recent national statistics indicate that approximately 42% of women in Norway have attained this level of education [55]. This educational overrepresentation in higher education may limit the generalisability of the findings to the broader population.

Three of the authors are licensed chiropractors with extensive clinical experience in treating women with musculoskeletal conditions, including PPGP. All authors entered the study with prior understanding of PPGP, which may be considered both an asset and a potential source of bias. This background facilitated the development of relevant interview questions and a deeper contextual understanding of participants’ experiences. At the same time, the risk of unintentionally shaping the interviews or interpretations was acknowledged. To address this, the analysis was conducted with methodological transparency and reflexivity. Texts were analysed independently by the authors and then collaboratively discussed until consensus was reached, enhancing the dependability and confirmability of the findings. Rich descriptions of the research process and analytical approach are provided, along with illustrative participant quotes, to further support the study’s trustworthiness and allow readers to follow the progression from data to interpretation.

## Conclusion

This study extends current understanding and adds novel insight by demonstrating how persistent PPGP is experienced not only as a physical condition, but as a multidimensional challenge that shapes women’s identity, caregiving roles, and engagement in everyday life during pregnancy. The voices of women emphasise the value of being acknowledged and validated by healthcare professionals and point to the importance of coordinated support across clinical, social, and workplace settings. Our findings underscore the need for a holistic, person-centred approach to PPGP in clinical care, one that accounts for the emotional, social, and occupational burdens that often accompany physical pain. Improving professional awareness and ensuring access to appropriate workplace accommodations may help mitigate long-term functional decline and enhance women’s ability to maintain autonomy and work participation during pregnancy. Future research should focus on developing and evaluating patient-informed strategies for managing daily symptoms. A clearer understanding of what women themselves identify as supportive will be crucial in designing targeted interventions that promote sustained well-being throughout pregnancy.

## Supplementary Information

Supplementary Material 1.

Supplementary Material 2.

## Data Availability

The datasets generated and/or analysed during the current study are not publicly available due to the sensitive nature of the qualitative data and the risk of compromising participant privacy. Participants did not consent to data sharing beyond the research team. Therefore, the data cannot be made publicly accessible. Further inquiries regarding the dataset can be directed to the corresponding author, subject to appropriate ethical considerations and institutional approval.

## Abbreviations

PPGP: Pregnancy-related Pelvic Girdle Pain
PGP-NOR: Pelvic Girdle Pain in a NORwegian cohort
PGQ: Pelvic Girdle Questionnaire
ANC: AntenatalCcare
eGP: General Practitioner
GA: Gestational Age
HRQoL: Health-Related Quality of Life
LKB: Landsforeningen for kvinnelig bekkenleddshelse (Norwegian female pelvic health association)
GDPR: General Data Protection Regulation
COREQ: Consolidated Criteria for Reporting Qualitative Research
SD: Standard Deviation
