# Supplementary material for: Observing life from the sideline – a qualitative study on experiences of living with pregnancy-related pelvic girdle pain
Source: BMC Pregnancy Childbirth. 2026 Feb 4;26:231. doi: 10.1186/s12884-026-08724-y (PMC12964835; doi:10.1186/s12884-026-08724-y)
Supplement: Supplementary file 2 — Supplementary Material 2. [file 12884_2026_8724_MOESM2_ESM.docx]

Appendix 1: Interview guide

Can you describe what it's like for you to have pelvic girdle pain?
Describe your everyday life with pelvic girdle pain.
How do others react when you tell them that you have pelvic girdle pain?
What do you think people who don't have pelvic girdle pain think about it?
Do you feel that the healthcare system (general practitioner/midwife/physiotherapist/health center) acknowledges your challenges with pelvic girdle pain?
What do you think would be helpful for you in your situation?
What do you do to relieve your pelvic girdle pain?
Have you received advice and information about pelvic girdle pain, and what advice was given? Who gave you the advice/information?
What did you know about pelvic girdle pain before pregnancy?
Do you think there is enough expertise in the healthcare system about pelvic girdle pain?
If no, what is missing?
Which advice has worked, and which has not, in your daily life?
Have you been examined for pelvic girdle pain? By whom?
Have you been recommended treatment for pelvic girdle pain? If yes, by whom?
Have you received/had treatment for pelvic girdle pain? If yes, from whom?
How did you experience the treatment you received? (What was good? What was bad?)
What are your thoughts about the future?
